# Association between Anticholinergic Burden and Constipation: A Systematic Review

**DOI:** 10.3390/healthcare9050581

**Published:** 2021-05-13

**Authors:** Héctor Rodríguez-Ramallo, Nerea Báez-Gutiérrez, Elena Prado-Mel, Eva Rocío Alfaro-Lara, Bernardo Santos-Ramos, Susana Sánchez-Fidalgo

**Affiliations:** 1 Pharmacy Department, Hospital Universitario Virgen del Rocío, 41013 Seville, Spain; hector.rodriguez.sspa@juntadeandalucia.es (H.R.-R.); Nerea.baez.sspa@juntadeandalucia.es (N.B.-G.); elena.prado.sspa@juntadeandalucia.es (E.P.-M.); Bernardo.santos.sspa@juntadeandalucia.es (B.S.-R.); 2 Department of Preventive Medicine and Public Health, Universidad de Sevilla, 41009 Seville, Spain; fidalgo@us.es

**Keywords:** anticholinergic burden, anticholinergic scale, anticholinergic drugs, constipation

## Abstract

The association between anticholinergic burden and constipation is not well defined and documented; for this reason, a systematic review was carried out in five databases (Medline, Embase, Cochrane Central Register of Controlled Trials, CINAHL, and Scopus), including studies assessing the correlation between anticholinergic burden, and constipation between January 2006 and December 2020. Data extraction was conducted independently by two researchers. Abstracts and titles were reviewed to determine eligibility for review with eligible articles read in full. From 2507 identified articles, 11 were selected for this review: six cross-sectional studies, four retrospective cohort studies, and a post hoc analysis of a randomized clinical trial. Overall, nine studies reported at least one statistical association between anticholinergic burden and constipation, finding 13 positive results out of 24 association measurements. A total of 211,921 patients were studied. The association between constipation and anticholinergic burden could be demonstrated in studies including 207,795 patients. Most studies were not designed to find differences in constipation prevalence and did not adjust the results by confounding factors. Our findings suggest that a correlation between anticholinergic burden and constipation exists. Higher quality-evidence studies are needed, including analysis that considers confounding factors, such as other non-pharmacological causes of constipation.

## 1. Introduction

Anticholinergic drugs are frequently prescribed for several indications: overactive bladder, gastrointestinal disorders, and bradycardia. Moreover, many drugs not recognized as anticholinergics, such as antidepressants, bronchodilators, skeletal muscle relaxants, and antispasmodics, often show anticholinergic activity. The estimated prevalence of anticholinergic drug exposition has been described as 50% in older patients; the usage of anticholinergic drugs appears to increase with age [1].

The cumulative effect of taking multiple anticholinergic drugs is defined as anticholinergic burden [2]. There is currently no standard for measuring this anticholinergic burden. However, several anticholinergic scales have been developed to estimate it [3,4,5,6,7,8]. Most scales assign an anticholinergic score to the drugs considered with anticholinergic activity. The final anticholinergic burden is the sum of the score for each drug that a patient receives.

The main use of quantifying anticholinergic burden is to estimate the risk of anticholinergic-related adverse effects. Several studies have established a relationship between increased anticholinergic burden and the appearance of different adverse effects. Most of these studies have focused on central adverse effects such as confusion, cognitive impairment, falls, and delirium [4,5,8,9]. Recent studies have been able to find a relationship between anticholinergic burden and length of hospital stay, the number of hospitalizations, and even mortality [10,11,12]. However, the appearance of peripheral adverse effects and specifically the relationship between anticholinergic burden and constipation have been less reported [4,10].

Constipation is a common gastrointestinal disorder. Its definition is based on subjective factors and symptoms [13,14]. The prevalence of constipation varies widely depending on sex, age, comorbidities, medication consumption, lack of adequate physical activity, lack of proper diet, and geographical location [15,16]. In adults, the estimated prevalence of constipation is 2% to 27% [16,17]. A high prevalence of constipation has been described in women, older adults, and institutionalized patients [18].

Constipation may hinder the functional status and quality of life of patients [19]. A systematic review found that patients with constipation showed an impaired health-related quality of life (HRQoL) compared with healthy patients. This effect was consistent on both mental and physical components of QoL [20]. This disorder may decrease productivity [21] and induce patients to spend on laxatives, expenses that reach millions of dollars just in the United States [22,23]. Constipation also increases the risk of hospitalization [24,25] and has been found as a risk factor for fecal impaction [26], a remarkable cause of hospitalization, morbidity, and mortality among older patients [24,27].

This systematic review was designed to answer the PICOS question: (Population), any population; (Intervention), drugs classified as having anticholinergic activity by an anticholinergic scale; (Comparison), none to low anticholinergic burden vs. medium to high anticholinergic burden; (Outcomes), constipation; (Study design), observational, interventional or cross-sectional studies.

Based on the foregoing, the primary objective of this systematic review was to identify the available evidence on the degree of association between anticholinergic medications, as measured by different scales, and constipation.

The secondary objectives were to identify the anticholinergic scales with the highest level of evidence supporting the existence of this association, to estimate the prevalence of constipation in patients treated with anticholinergic drugs, and analyze the homogeneity of the results among the different anticholinergic scales in the published articles.

## 2. Materials and Methods

### 2.1. Information Sources

A systematic review was performed on scientific literature published between 1 January 2006 and 1 December 2020, in the MEDLINE, EMBASE, Cochrane Central Register of Controlled Trials, CINAHL, and SCOPUS databases. The search was conducted following the “Preferred Reporting Elements for Systematic Reviews and Meta-analysis” (PRISMA) guide [28] (Supplementary File S1). The search was performed in December 2020.

Articles published before 2006 were excluded due to most anticholinergic scales being designed and published after this date.

### 2.2. Search

The research strategy was developed based on the PICOS question: A mixture of MeSH terms and controlled text were used. A detailed search strategy of all databases can be found in Supplementary File S2.

The reference list of each article was manually reviewed, and a citation analysis was performed to identify articles missed by the search strategy.

### 2.3. Study Eligibility Criteria

#### 2.3.1. Studies Inclusion Criteria

Articles that measure anticholinergic burden with any anticholinergic scales designed to estimate anticholinergic burden [3,4,5,6,7,8] and its statistical association with constipation. 

Despite not performing an anticholinergic burden calculation, articles that compare the prevalence of constipation in patients with/without anticholinergic drugs. An anticholinergic scale must be used to define the drugs considered to have an anticholinergic effect, even when the anticholinergic burden was not estimated.

No language, age, population, and setting restrictions were applied.

#### 2.3.2. Exclusion Criteria

- The full text was not available.
- Constipation as a composite variable.
- Narrative reviews, letters to the editor, and conference summaries.
- Articles that measured only acute constipation.
- The association magnitude was not disclosed.

### 2.4. Study Selection

The study selection was performed in pairs (HR and NB), first by title and abstract. For those articles in which this was not enough to verify compliance with the criteria, a third reviewer (SF) was in charge of resolving any disagreement. Reasons for exclusion on the full-text articles assessed for eligibility can be found in Supplementary File S3.

### 2.5. Assessment of Articles Quality

The quality of observational articles was assessed with the Joanna Briggs Institute (JBI) Critical Appraisal tools for cohort, case-control, and cross-sectional studies [29]. Randomized controlled trials quality were assessed by RoB 2.0 [30]. The quality outcomes of each included study are shown in Supplementary File S4.

### 2.6. Data Collection

Full-text copies of included articles were reviewed, and data were extracted and entered into a structured Microsoft Excel, Microsoft Corporation (Redmond, WA, USA) database. The variables collected were:

- Study characteristics: Study design (Interventional, observational, cross-sectional studies), country, the number of patients included, study duration.
- Patient characteristics: the setting of the population included (hospital, home-dwelling patients, nursing facilities inmates, community group homes, or patients from health insurance databases), average age or age limits for inclusion on each study, sex.
- Variables related to constipation: constipation diagnostic methods, number and percentage of patients diagnosed with constipation, patient’s physical activity, patient´s fluid and fiber intakes, polypharmacy rates, opioids use prevalence and patient´s diagnoses that may contribute to constipation: obstructive digestive diseases, mechanical causes of constipation, neurologic disorders such as Parkinson’s disease and multiple sclerosis, myogenic disorders, and enteric neuropathies.
- Anticholinergic activity data: number and percentage of patients treated with anticholinergic drugs and the anticholinergic scale used.
- Data about the association between constipation and anticholinergic burden: positive (significant data) or negative association (non-significant data) according to association measure (OR, *p*-value, RR, Hazard ratio, etc.), anticholinergic drugs that most frequently were associated with constipation.

## 3. Results

**Figure 1:**
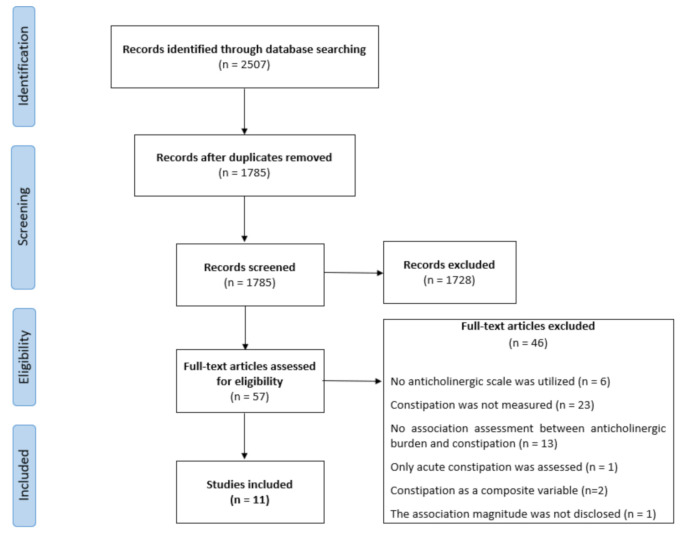
Prisma flow-diagram.

The search identified 2507 studies. After the first review by title or abstract and removing duplicates, 57 articles were selected for a full-text review. Finally, 11 articles were included [10,31,32,33,34,35,36,37,38,39,40]. Most articles were excluded because constipation was not measured (Figure 1). 

The review of the reference lists and citation analyses assessed 454 studies. No additional studies that met the inclusion criteria were identified. 

### 3.1. General Characteristics of the Included Studies

Included articles had remarkable differences between their aims: the majority of them aimed to estimate the possible relationship between the use of anticholinergic drugs and adverse outcomes [10,31,33,36,37,38,39,40].

Allen et al., 2017 [34], aimed to determine the differences in prevalence, clinical characteristics, and treatment among patients with or without constipation within a sample of patients whose treatment included opioids. Briet et al., 2017 [35], aimed to design and validate an anticholinergic scale. Wawruch et al., 2011 [32], aimed to evaluate the use of drugs with anticholinergic properties in older patients and identify risk factors that increase the patient’s chance of being given such medications.

Ten out of the eleven included articles reported were observational studies, six were cross-sectional [33,35,36,37,38,39], four were retrospective cohort studies [10,32,34,40], and the remaining article reported a post-hoc analysis of a controlled clinical trial [31]. 

Overall, 211,921 people were included in the 11 articles; 1871 were hospital inpatients, 3512 were home-dwelling patients, 15,225 resided in care homes or equivalents, and 191,306 were studied from a National Health Insurance database. For seven patients, the setting was not defined. Five and six studies included exclusively adult patients (>18 years) and older patients (>65 years), respectively (Table 1). 

### 3.2. Constipation Prevalence and Anticholinergic Exposure

The methods used to diagnose a patient with constipation varied remarkably. Four studies used laxatives as an indicator of constipation [10,35,36,37]. In three studies, patients were classified as constipated by clinicians’ assessment [31,32,39]. One study used straight questions to the patients with a clinical diagnosis to determine if they suffered from constipation [38]. One study classified patients as constipated by reviewing the diagnostic codes in emergency department visit claims [40]. Two studies used a combination of the above-explained methods [33,34].

The most frequently prescribed pharmacological groups in the included studies were: opioids [32,33,35], antipsychotics [34,36,38], and antiepileptics [39]. Four studies did not specify which anticholinergic drugs were prescribed on the studied patients [10,37,40] (Table 2).

### 3.3. Alternative Causes of Constipation

The patient’s physical activity, fiber, and fluid intake were not measured in any included studies. A low physical activity could be assumed for hospital and palliative patients [31,32,38,39]. Polypharmacy rates were higher in patients who received anticholinergic drugs in several studies [39,40]. A higher number of drugs in patients with anticholinergic burden was also described in one study [10]. 

The studies included did not report comparative data on the rates of opioids usage and causes of constipation. Among diagnoses related to constipation, only one study reported data on the prevalence of Parkinson’s disease on the studied population [32] (Supplementary Table S1).

### 3.4. Methods for Measuring Anticholinergic Burden

A total of 10 anticholinergic scales were used on the studies selected for this review [3,4,5,6,7,8,35,41,42,43]. Three studies used several anticholinergic scales to estimate anticholinergic burden: Mayer et al., 2017 [36], calculated anticholinergic drug exposure with nine different scales. Sevilla-Sánchez et al., 2018 [39], compared the anticholinergic drug exposure using two different scales: the Anticholinergic Drug Scale (ADS) [7] and the Drug Burden Index (DBI) [8]. Finally, in the study by Wawruch et al., 2011 [32], Anticholinergic Risk Scale (ARS) [4] and the Clinician-Rated Anticholinergic Scale (CRAS) [42] were used. The other eight studies used a single scale to measure anticholinergic activity. 

Some studies performed modifications in the anticholinergic scales by increasing the number of included anticholinergic drugs. O’Dwyer et al., 2016, and Hwang et al., 2019 [33,40], chose to include anticholinergic drugs available in Ireland and Korea, respectively. Moreover, Hwang et al., 2019 [40], used the ARS score [4] adjusted through the defined daily dose (DDD) value assigned by the WHO [44,45].

Allen et al., 2017 [34], and Wawruch et al., 2011 [32], in order to avoid overestimating the anticholinergic burden, only included medications with a score of ≥2.

Mayer et al., 2017 [36], decided to estimate the relationship between the anticholinergic burden and constipation by combining the results of the utilized nine anticholinergic scales.

### 3.5. Association between Anticholinergic Burden and Constipation

The eleven studies included in this review performed 24 association measurements. Overall, 9 out of 11 studies reported a statistical association between anticholinergic burden and constipation, finding a total of 13 positive results out of 24 association measurements according to different scales (Table 3).

An association between anticholinergic burden and constipation was observed in studies that included 207,795 patients. 

Most of the included studies, 10 out of 11, grouped patients according to anticholinergic burden scores, analyzing the differences in adverse event prevalence. On the other hand, Allen et al., 2017 [34], chose to group patients according to whether they had constipation or not. Then, they looked for differences between both groups, finding significant statistical distinctions when dealing with anticholinergic burden, thus presenting higher values in patients with constipation.

### 3.6. Association Results by Anticholinergic Scale

Table 4 highlights the fact that the ARS [4] was used to evaluate the anticholinergic burden in most patients included in this review. This scale also presented the highest number of favorable results when estimating a relationship between the anticholinergic burden and constipation. 

## 4. Discussion

To our knowledge, this is the first systematic review searching for the degree of association between anticholinergic medications, as measured by different anticholinergic scales and constipation. 

There is an extensive number of studies looking for a correlation between anticholinergic burden and central adverse effects in the literature. However, this review shows the lack of studies testing a relationship between the anticholinergic burden and constipation.

Most of the articles and reviews that seek to establish a relationship between anticholinergic adverse effects and anticholinergic burden focus on older patients, with the exception of 4 studies [33,34,35,38] which analysed data from patients younger than 65 years. In contrast with other published reviews searching for the relation between the anticholinergic burden and adverse outcomes, the authors of this review chose not to limit the search to older patients. This decision was made due to the small number of articles that included constipation as a peripheral adverse event in the previously published reviews [11,46,47,48]. Even without setting an age restriction, the number of studies that met the inclusion criteria for this review was low. 

On the other hand, previous reviews had reported different scales as the most frequently validated tools to assess an association between anticholinergic burden and central adverse events. In this way, some authors described the Anticholinergic Cognitive Burden Scale (ACB) [3] and the DBI [8] as the anticholinergic scales most frequently used [11,46,47]. In contrast with the scales described for central adverse effects, the ARS was the most frequently used scale to assess the association between constipation and anticholinergic burden among the studies included in this review most frequently utilized.

In the studies that met the criteria for this review, we found great diversity in methodology and results, including the type of patients studied, the diagnostic criteria of constipation, constipation prevalence, and the different scales used to evaluate the anticholinergic risk. Considering this diversity, it is not surprising to observe differences in the use of anticholinergic drugs and constipation.

As observed by Fox et al., 2011 [1], medications with possible anticholinergic properties account for up to 50% of all drugs prescribed for older adults. Our observations are consistent with this assumption; most included studies described an anticholinergic exposure above 50%. 

The constipation diagnosis method chosen has an undeniable impact on the observed constipation prevalence. This is especially striking in Hwang et al., 2019 [40], which described a constipation prevalence of 0.5% even when 63.5% of patients were exposed to anticholinergic drugs. This low constipation prevalence is probably a consequence of the diagnosis method, as they used emergency department visits data, which may differ in routine clinical settings or constipation assessed from laxative prescriptions. 

Previous studies have shown that “the general population’s perceptions of constipation differ significantly from those of general practitioners and specialists, and there is a limited agreement between perceptions of constipation and Rome IV criteria” [49]. Using the symptoms reported by the patients without using comprehensive clinical criteria may result in an overestimation of constipation [50]. Patients tend to define constipation through symptoms that would not necessarily be diagnosed as such by a physician.

As stated, constipation may be associated with diverse conditions and habits; these may act as confounding factors when establishing an association with the anticholinergic burden. When searching for these variables, most studies were not designed to identify the causes of constipation but instead looked at its relationship with the anticholinergic burden within its secondary endpoints. Furthermore, in the rare cases where some confounding factors were collected [31,32,33,39,40], the data were not expressed separately by patients with/without constipation, which makes it impossible to search for differences between cohorts.

The only article included in this review that compared the use of anticholinergic drugs measured with several scales [39] found a significantly higher usage of anticholinergic drugs when they were assessed with the ADS [7] compared with the DBI [8]. These two scales are considered the most similar when used to assess whether drugs contribute to anticholinergic burden [51]. This result highlights the differences in the drugs considered to add to the anticholinergic burden by the different scales.

Differences in drugs with anticholinergic properties used in different countries may have caused the absence of statistical significance of the results in some cases. In the studies that include scales modifications to add anticholinergic drugs frequently used in their environment [33,40], a positive relationship between constipation and the anticholinergic burden was obtained. This suggests that scale modifications are necessary in different settings and countries in order to better estimate the anticholinergic burden.

When analyzing the included studies by study design, the cross-sectional studies included in this review obtained great diversity in their results. However, all higher level of evidence studies, consisting of four retrospective cohort studies, described a correlation between anticholinergic burden and constipation. Precaution is needed when interpreting the negative result of the remaining study due to its nature as a post-hoc analysis of a clinical trial. These results suggest that a relationship between anticholinergic burden and constipation exists, as the studies on the higher levels of evidence available appear to suggest it.

The authors performed different analyses when studying the relationship between anticholinergic burden and constipation. In studies searching for differences in constipation, prevalence between patients with high and low anticholinergic burden showed the most positive results [33,35,37,38,39,40]. Therefore, low values of anticholinergic burden appear not to increase the risk of constipation; this contrasts with a high anticholinergic burden which did seem to increase this risk. This hypothesis is consistent with the previously described association between constipation and anticholinergic activity [52].

This review had several strengths: An electronic search was conducted in five different databases to identify all potential studies that met the strict predefined eligibility criteria. A systematic approach was taken to describe and synthesize all findings of this review. This review aimed to find all possible evidence about an association between constipation and anticholinergic burden, which allowed us to identify several published studies which were not included in previous reviews.

This study had several limitations: most of the included articles were observational studies. Only a post hoc analysis of an interventional study complied with the inclusion criteria for this review. This limits the capacity to reach conclusions about the relationship between anticholinergic burden and constipation. Moreover, the clinical and methodological diversity among the studies did not allow to carry out a meta-analysis.

## 5. Conclusions

This systematic review suggests that a correlation between anticholinergic burden measured by different scales and constipation exists. Although only a few studies searching for this relationship have been published, there is great diversity on the anticholinergic scale used, the methods to assess constipation, and the type of population included.

Higher quality-evidence studies are needed, including analysis that considers confounding factors, such as other non-pharmacological causes of constipation. Future reviews may also explore strategies to minimize this common side effect of drugs with anticholinergic properties.

## Figures and Tables

**Table 1:** General characteristics of the included studies.

Study Year	Study Design	Population Setting (N)	Country	Age (Y)	Patients (N)	Duration (M)
Agar M. 2009	Post-hoc analysis of a randomized controlled trial	Home-dwelling: 89.0% (410) Nursing homes 6.5% (30)	Australia	71 ± 12 ^†^	461 §	24
Wawruch M. 2011	Retrospective cohort study	Hospital	Slovakia	78 ± 7 ^†^	1636	24
Kuang-Hua H.2012	Retrospective cohort study	National Health Insurance database	Taiwan	>65	72,556	12
Allen C. 2017	Retrospective cohort study	Nursing homes	United States	>18	6556	24
Hwang S. 2019	Retrospective cohort study	National Health Insurance database	Korea	75 ± 7 ^†^	118,750	3
O’Dwyer M. 2016	Cross-sectional study	Home-dwelling: 16.6% (122) Nursing homes: 83.4% (614)	Ireland	54 ± 9 ^†^	736	-
Briet J. 2017	Cross-sectional study	Psychiatric facility	France	50 ^‡^	7278	-
Mayer T. 2017	Cross-sectional study	Home-dwelling	Germany	73 ± 6 ^†^	2761	-
De Vreese L. P. 2018	Cross-sectional study	Nursing homes 57.6% (159) Home-dwelling 42.4% (117)	Italy	55 ± 8 ^†^	276	-
O’Connell J. 2018	Cross-sectional study	Nursing homes: 84.9% (574) Home-dwelling: 15.1% (102)	Ireland	>40	676	-
Sevilla-Sánchez D. 2018	Cross-sectional study	Hospital	Spain	87 ± 5 ^†^	235	-

^†^ Average (Standard deviation). ^‡^ Median. ^§^ Analysis included only 304 patients. Female %, Percent of female patients in the studied population. M, Months.

**Table 2:** Constipation and anticholinergic activity data.

Study Year	Patients (N)	Assessment Method for Constipation	Constipation Prevalence (N)	Anticholinergic Exposure (N)	Most Prescribed Drugs
Agar M. 2009	461	Clinical assessment	No Data	No Data	Opioids
Wawruch M. 2011	1636	Clinical assessment	8.0% (131)	Admission: 10.5% (172) ^‡^ Discharge: 13.6% (223) ^‡^	Opioids
Sevilla-Sánchez D. 2018	235	Clinical assessment	62.1% (146)	ADS: 93.6% (220) DBI: 82.1% (193)	No Data
O’Dwyer M. 2016	736	Clinical assessment and laxative prescriptions	No Data	70.1% (516)	Antipsychotics
Allen C. 2017	6556	Clinical charts andlaxative prescriptions	8.9% ^†^	73.4% (4811)	Opioids
Briet J. 2017	7278	Laxative prescriptions	48.2% (3509)	97.2% (7077)	Antipsychotics
Mayer T. 2017	2761	Laxative prescriptions	1.5% (41)	45.6% (1258)	No Data
De Vreese L. P. 2018	276	Laxative prescriptions	10.5% (29)	35.5% (98)	Antipsychotics
Kuang-Hua H. 2012	72,556	Laxative prescriptions	7.9% (5742)	75.7% (54,888)	No Data
O’Connell J. 2018	676	Patient´s self-reports	38.4% (257)	78.6% (532)	Antiepileptics
Hwang S. 2019	118,750	Diagnostic codes in emergency department visit claims	0.5% (567)	36.5% ^†^	No Data

^†^ Prevalence/exposure was estimated from the entire population of the database; the cohort study was carried with a population sample. ^‡^ Measured with a combination of the anticholinergic scales. No Data: no information about this variable could be found in the original paper.

**Table 3:** Association between anticholinergic burden and constipation.

Study Year	Patient’s Groups Compared When Looking for a Correlation	Anticholinergic Scale	Association §	Association Magnitude §
Wawruch M. 2011	Anticholinergic burden; 0 vs. >0 *	Anticholinergic Risk Scale ^†^	+	OR: 1.91 (1.18–3.11)
		Clinician-Rated Anticholinergic Scale ^†^		
Kuang-Hua H. 2012	Anticholinergic burden; 0 vs. >0 *	Anticholinergic Risk Scale	+	OR: 1.87 (1.72–2.03)
Hwang S. 2019	Anticholinergic burden; 0 vs. ≥2	Anticholinergic Risk Scale ^†^	+	HR: 1.65 (1.35–2.02)
Mayer T. 2017	Anticholinergic burden; 0 vs. >0 *	Anticholinergic Risk Scale	+	OR: 1.47 (p = 0.02)
		Anticholinergic Burden Classification	+	OR: 1.58 (p < 0.001)
		Cancelli’s Anticholinergic Burden Scale	+	OR: 1.58 (*p* < 0.001)
		Chew’s list	+	OR: 1.57 (*p* = 0.002)
		Anticholinergic Drug Scale	-	OR: 1.24 (*p* = 0.17)
		Clinician-Rated Anticholinergic Scale	-	OR: 1.47 (*p* = 0.08)
		Drug Burden Index	-	OR: 1.14 (*p* = 0.45)
		Anticholinergic Loading scale	-	OR: 1.14 (*p* = 0.45)
		Anticholinergic Cognitive Burden Scale	-	OR: 1.06 (*p* = 0.77)
‘O’Dwyer M. 2016	Anticholinergic burden; 0 vs. 1–4 vs. >5	Anticholinergic Cognitive Burden Scale ^†^	+	χ2: *p* < 0.001
Allen C. 2017	Patients with and without constipation	Anticholinergic Cognitive Burden Scale	+	χ2: *p* < 0.001
De Vreese L. P. 2018	Anticholinergic burden; 0 vs. >3	Anticholinergic Cognitive Burden Scale	+	χ2: *p* = 0.003
O’Connell J. 2018	Anticholinergic burden; 0 vs. (0–1) ‡	Drug Burden Index	-	OR: 1.28 (0.64–2.53)
	Anticholinergic burden; 0 vs. ≥1		-	OR: 1.68 (0.90–3.12)
Sevilla-Sánchez D. 2018	Anticholinergic burden; 0 vs. >0 *	Drug Burden Index	+	χ2: *p* = 0.03
	Anticholinergic burden; 0 vs. 1–2 vs. >2		-	χ2: *p* = 0.11
	Anticholinergic burden; 0 vs. >0 *	Anticholinergic Drug Scale	-	χ2: *p* = 0.46
	Anticholinergic burden; < 3 vs. ≥3		-	χ2: *p* = 0.92
Agar M. 2009	Anticholinergic burden; 0 vs. >0	Clinician-Rated Anticholinergic Scale ^†^	-	OR: 1.05 (0.98–1.12)
Briet J. 2017	Anticholinergic burden; ≤5 vs.>5	Anticholinergic impregnation scale	+	OR: 2.03 (1.81–2.28)

* This association assessment compared patients who were prescribed anticholinergic drugs vs. patients without said drugs. † The Anticholinergic scale was modified for this study. ‡ Low Anticholinergic burden. § Multivariate analysis data were collected preferably over univariate analysis. (+) Association present, (-) Association absent. OR: Odds ratio. χ^2^: Pearson’s chi-squared test. HR: Hazard ratio.

**Table 4:** Synthesis of studies and association measurements by anticholinergic scale.

Anticholinergic Scale	Studies	Association Assessments	Association Present (+)		Association Absent (-)	
			Association Assessments	Patients Involved	Association Assessments	Patients Involved
Anticholinergic Risk Scale †	4	4	4	195,703	0	0
Anticholinergic Cognitive Burden Scale	4	4	3	7568	1	2761
Clinician-Rated Anticholinergic Scale †	3	3	1	1636	2	3222
Drug Burden Index	3	5	1	235	4	4113
Anticholinergic Drug Scale	2	3	0	0	3	2555
Anticholinergic impregnation scale	1	1	1	7278	0	0
Cancelli’s Anticholinergic Burden Scale	1	1	1	2761	0	0
Chew’s list	1	1	1	2761	0	0
Anticholinergic Burden Classification	1	1	1	2761	0	0
Anticholinergic Loading scale	1	1	0	0	1	2761

^†^ As Wawruch M., 2011, combined the Anticholinergic Risk scale and the Clinician-Rated Anticholinergic Scale, the association assessments were counted for both scales.

## Data Availability

All data related to this study are available as Supplementary Files.

## Supplementary Materials

The following are available online at https://www.mdpi.com/article/10.3390/healthcare9050581/s1. Supplementary Table S1; Alternate causes of constipation, Supplementary File S1; Prisma Checklist, Supplementary File S2: Search strategies, Supplementary File S3: Full-text articles excluded and causes of exclusion, Supplementary File S4: Quality assessment of included studies.
