# Supplementary material for: Association between Anticholinergic Burden and Constipation: A Systematic Review
Source: Healthcare (Basel). 2021 May 13;9(5):581. doi: 10.3390/healthcare9050581 (PMC8153334; doi:10.3390/healthcare9050581)
Supplement: Supplementary file 1 [file healthcare-09-00581-s001.zip › healthcare-1174593 Appendix and Supplementary author proofreading done/Supplementary File S2.pdf]

Association between anticholinergic burden and constipation: a systematic review.

Supplemental file S2. Search strategies.

Medline

#1. Anticholinergic OR Cholinergic Antagonists [MeSH term] OR Muscarinic Antagonists [MeSH term]

#2. Burden OR Scale OR Load

#3. Constipation [MeSH term] OR Intestinal Disorders OR Laxatives [MeSH term] OR Cathartics [MeSH term] OR "Drug-Related Side Effects and Adverse Reactions" [MeSH term] OR Side Effects [MeSH term] OR Adverse Drug Event [MeSH term] OR Adverse Effects OR Drug Toxicity [MeSH term] OR Adverse Outcomes OR Risk Factors [MeSH term]

#4. (#1 AND #2) AND #3

#5. Limit "humans"

#6. Limit Jan 2006 to Dec 2020

EMBASE

#1. Anticholinergic OR 'cholinergic antagonists' OR 'Muscarinic Antagonists'

#2. Burden OR Scale OR load

#3. Constipation OR 'intestinal disorders' OR laxatives or Cathartics OR 'Drug-Related Side Effects and Adverse Reactions' OR 'Side Effects' OR 'Adverse Drug Event' OR 'Adverse Effects' OR 'Drug Toxicity' OR 'adverse outcomes' OR 'risk factors'

#4. #1 AND #2

#5. #3 AND #4

#6. Limit “humans”

#7. Limit Jan 2006 to Dec 2020

Cochrane

#1. Anticholinergic OR Cholinergic Antagonists OR Muscarinic Antagonists

#2. Burden OR Scale OR Load

#3. Constipation OR intestinal disorders OR laxatives or Cathartics or (Drug-Related Side Effects and Adverse Reactions) or Side Effects OR Adverse Drug Event OR Adverse Effects OR Drug Toxicity OR adverse outcomes OR risk factors

#4. #1 AND #2

#5. #3 AND #4

#6. in Title Abstract Keyword

#7. Limit Jan 2006 to Dec 2020

SCOPUS

#1. Anticholinergic OR 'cholinergic antagonists' OR 'Muscarinic Antagonists'

#2. Burden OR Scale OR load

#3. Constipation OR 'intestinal disorders' OR laxatives or Cathartics OR 'Drug-Related Side Effects and Adverse Reactions' OR 'Side Effects' OR 'Adverse Drug Event' OR 'Adverse Effects' OR 'Drug Toxicity' OR 'adverse outcomes' OR 'risk factors'

#4. #1 AND #2

#5. #3 AND #4

#6. Limit “humans”

#7. Limit Jan 2006 to Dec 2020

Cinahl

#1. Anticholinergic OR Cholinergic Antagonists OR Muscarinic Antagonists

#2. Burden OR Scale OR Load

#3. Constipation OR intestinal disorders OR laxatives or Cathartics or (Drug-Related Side Effects and Adverse Reactions) or Side Effects OR Adverse Drug Event OR Adverse Effects OR Drug Toxicity OR adverse outcomes OR risk factors

#4. #1 AND #2

#5. #3 AND #4

#6. Limit Jan 2006 to Dec 2020
