# Supplementary material for: Association between Anticholinergic Burden and Constipation: A Systematic Review
Source: Healthcare (Basel). 2021 May 13;9(5):581. doi: 10.3390/healthcare9050581 (PMC8153334; doi:10.3390/healthcare9050581)
Supplement: Supplementary file 1 [file healthcare-09-00581-s001.zip › healthcare-1174593 Appendix and Supplementary author proofreading done/Supplementary File S3.pdf]

Association between anticholinergic burden and constipation: a systematic review.

Supplemental file S3. Full-text articles excluded and causes of exclusion.

Reference	Exclusion criteria
Inkeri NM, Karjalainen M, Haanpää M, et al. Anticholinergic drug use and its association with self-reported symptoms among older persons with and without diabetes. J Clin Pharm Ther. 2019;44(2):229–35.	The association magnitude was not disclosed.
Hughes J, Puangsombat J, Roberts M. The association between anticholinergic load and cognitive performance, elevated blood pressure and the presence of peripheral anticholinergic side effects. Australasian Med J 2009;1:1—27	No anticholinergic scale was utilized.
Carnahan RM, Lund BC, Perry PJ, Pollock BG, Culp KR. The Anticholinergic Drug Scale as a measure of drug-related anticholinergic burden: associations with serum anticholinergic activity. J Clin Pharmacol. 2006 Dec;46(12):1481-6.	Constipation was not measured
Plaschke K, Thomas C, Engelhardt R, Teschendorf P, Hestermann U, Weigand MA, Martin E, Kopitz J. Significant correlation between plasma and CSF anticholinergic activity in presurgical patients. Neurosci Lett. 2007 Apr 24;417(1):16-20.	No anticholinergic scale was utilized.
Nebes RD, Pollock BG, Halligan EM, Kirshner MA, Houck PR. Serum anticholinergic activity and motor performance in elderly persons. J Gerontol A Biol Sci Med Sci. 2007 Jan;62(1):83-5	Constipation was not measured
Ancelin ML, Artero S, Portet F, Dupuy AM, Touchon J, Ritchie K. Non-degenerative mild cognitive impairment in elderly people and use of anticholinergic drugs: longitudinal cohort study. BMJ. 2006 Feb 25;332(7539):455-9.	Constipation was not measured
Ness J, Hoth A, Barnett MJ, Shorr RI, Kaboli PJ. Anticholinergic medications in community-dwelling older veterans: prevalence of anticholinergic symptoms, symptom burden, and adverse drug events. Am J Geriatr Pharmacother. 2006 Mar;4(1):42-51	No anticholinergic scale was utilized.
Landi F, Russo A, Liperoti R, Cesari M, Barillaro C, Pahor M, Bernabei R, Onder G. Anticholinergic drugs and physical function among frail elderly population. Clin Pharmacol Ther. 2007 Feb;81(2):235-41	No anticholinergic scale was utilized.
Salahudeen MS, Nishtala PS, Duffull SB. The Influence of Patient Characteristics on Anticholinergic Events in Older People. Dement Geriatr Cogn Dis Extra. 2016 Jan 6;5(3):530-41.	Constipation as a composite variable

Hilmer SN, Mager DE, Simonsick EM, Cao Y, Ling SM, Windham BG, Harris TB, Hanlon JT, Rubin SM, Shorr RI, Bauer DC, Abernethy DR. A drug burden index to define the functional burden of medications in older people. Arch Intern Med. 2007 Apr 23;167(8):781-7.	Constipation was not measured
Aalto UL, Finne-Soveri H, Kautiainen H, Roitto HM, Öhman H, Pitkälä KH. Use of Anticholinergic Drugs According to Various Criteria and Their Association With Psychological Well-Being and Mortality in Long-Term Care Facilities. J Am Med Dir Assoc. 2019 Sep;20(9):1156-1162	No anticholinergic scale was utilized.
Weglinski L, Manceau P, Thomas-Pohl M, Le Breton F, Amarenco G. Évaluation prospective de l'impact des anticholinergiques sur la sécheresse buccale et oculaire chez 35 patients atteints de sclérose en plaque avec hyperactivité vésicale neurogène [Prospective evaluation of mouth and eye dryness induced by antimuscarinic drugs used for neurogenic overactive bladder in 35 patients with multiple sclerosis]. Prog Urol. 2017 Mar;27(4):253-260. French.	No anticholinergic scale was utilized.
Chougule A, Praharaj SK, Bhat SM, Sharma PSVN. Prevalence and Factors Associated With Clozapine-Related Constipation: An Observational Study. J Clin Psychopharmacol. 2018;38(1):42-46.	No association assessment between anticholinergic burden and constipation
Rudolph JL, Salow MJ, Angelini MC, McGlinchey RE. The anticholinergic risk scale and anticholinergic adverse effects in older persons. Arch Intern Med. 2008 Mar 10;168(5):508-13.	Constipation as a composite variable
Gouraud-Tanguy A, Berlioz-Thibal M, Brisseau J-M, Ould Aoudia V, Beauchet O, Berrut G, et al. [Analysis of iatrogenic risk related to anticholinergic effects using two scales in acute geriatric inpatient unit]. Geriatr Psychol Neuropsychiatr Vieil. 2012 Mar;10(1):27–32.	No association assessment between anticholinergic burden and constipation
Onda M, Imai H, Takada Y, Fujii S, Shono T, Nanaumi Y. Identification and prevalence of adverse drug events caused by potentially inappropriate medication in homebound elderly patients: a retrospective study using a nationwide survey in Japan. BMJ Open. 2015 Aug;5(8):e007581.	No association assessment between anticholinergic burden and constipation
Nguyen PV-Q, Pelletier L, Payot I, Latour J. The Delirium Drug Scale is associated to delirium incidence in the emergency department. Int psychogeriatrics. 2018 Apr;30(4):503–10.	No association assessment between anticholinergic burden and constipation
Montastruc F, Rouanet S, Gardette V, Rousseau V, Bagheri H, Montastruc J-L. Atropinic burden of prescriptions forms in patients with Alzheimer disease: a cross-sectional study in a French PharmacoVigilance Database. Eur J Clin Pharmacol. 2015 Jul;71(7):891–5.	No association assessment between anticholinergic burden and constipation
Chougule A, Praharaj SK, Bhat SM, Sharma PSVN. Prevalence and Factors Associated With Clozapine-Related Constipation: An Observational Study. J Clin Psychopharmacol. 2018 Feb;38(1):42–6.	No association assessment between anticholinergic burden and constipation
Lertxundi U, Isla A, Solinis MA, Domingo-Echaburu S, Hernandez R, Peral-Aguirregoitia J, et al. Anticholinergic burden in Parkinson's disease inpatients. Eur J Clin Pharmacol. 2015 Oct;71(10):1271–7.	No association assessment between anticholinergic burden and constipation

Yayla EM, Yavuz E, Bilge U, Keskin A, Binen E. Drugs with anticholinergic side-effects in primary care. Niger J Clin Pract. 2015;18(1):18–21.	No association assessment between anticholinergic burden and constipation
Geller EJ, Crane AK, Wells EC, Robinson BL, Jannelli ML, Khandelwal CM, et al. Effect of anticholinergic use for the treatment of overactive bladder on cognitive function in postmenopausal women. Clin Drug Investig. 2012;32(10):697–705.	No association assessment between anticholinergic burden and constipation
Rojo-Sanchís M, Vélez-Díaz-Pallarés M, Muñoz García M, et al. Reduction of anticholinergic burden in older patients admitted to a multidisciplinary geriatric acute care unit. Eur Geriatr Med. 2017;8(5–6):492–5.	No association assessment between anticholinergic burden and constipation
Sevilla-Sanchez D, Molist-Brunet N, Amblàs-Novellas J, Roura-Poch P, Espaulella-Panicot J, Codina-Jané C. Adverse drug events in patients with advanced chronic conditions who have a prognosis of limited life expectancy at hospital admission. *Eur J Clin Pharmacol*. 2017;73(1):79–89.	No association assessment between anticholinergic burden and constipation
Ivchenko A, Bodeker R-H, et al. Anticholinergic burden and comorbidities in patients attending treatment with trospium chloride for overactive bladder in a real-life setting: results of a prospective non-interventional study. BMC Urol [Internet]. 2018 Sep 14;18(1):N.	No association assessment between anticholinergic burden and constipation
Naples JG, Marcum ZA, Perera S, et al. Concordance Between Anticholinergic Burden Scales. J Am Geriatr Soc. 2015;63(10):2120-2124.	Constipation was not measured
Rojo-Sanchis AM, Velez-Diaz-Pallares M, Munoz Garcia M, Delgado Silveira E, Bermejo Vicedo T, Cruz Jentoft A. [Anticholinergic burden and delirium in elderly patients during acute hospital admission]. Rev Esp Geriatr Gerontol. 2016;51(4):217–20.	Constipation was not measured
McLarin PE, Peterson GM, Curtain CM, Nishtala PS, Hannan PJ, Castelino RL. Impact of residential medication management reviews on anticholinergic burden in aged care residents. Curr Med Res Opin. 2016;32(1):123–31.	Constipation was not measured
Bostock CV, Soiza RL, Mangoni AA. Associations between different measures of anticholinergic drug exposure and Barthel Index in older hospitalized patients. Ther Adv Drug Saf. 2013;4(6):235–45.	Constipation was not measured
Gnjidic D, Le Couteur DG, Abernethy DR, Hilmer SN. A pilot randomized clinical trial utilizing the drug burden index to reduce exposure to anticholinergic and sedative medications in older people. Ann Pharmacother. 2010 Nov;44(11):1725–32.	Constipation was not measured
Sathienluckana T, Unaharassamee W, Suthisisang C, Suanchang O, Suansanae T. Anticholinergic discontinuation and cognitive functions in patients with schizophrenia: a pharmacist-physician collaboration in the outpatient department. *Integr Pharm Res Pract*. 2018;7:161–171.	Constipation was not measured
Myint PK, Fox C, Kwok CS, Luben RN, Wareham NJ, Khaw KT. Total anticholinergic burden and risk of mortality and cardiovascular disease over	Constipation was not measured

10 years in 21,636 middle-aged and older men and women of EPIC-Norfolk prospective population study. *Age Ageing*. 2015;44(2):219–225.	
Desmarais JE, Beauclair L, Annable L, Bélanger MC, Kolivakis TT, Margolese HC. Effects of discontinuing anticholinergic treatment on movement disorders, cognition and psychopathology in patients with schizophrenia. *Ther Adv Psychopharmacol*. 2014;4(6):257–267.	Constipation was not measured
Kouladjian L, Gnjidic D, Chen TF, Mangoni AA, Hilmer SN. Drug Burden Index in older adults: theoretical and practical issues. Clin Interv Aging. 2014;9:1503–15.	Constipation was not measured
Kersten H, Molden E, Tolo IK, Skovlund E, Engedal K, Wyller TB. Cognitive Effects of Reducing Anticholinergic Drug Burden in a Frail Elderly Population: A Randomized Controlled Trial. Journals Gerontol Ser A Biol Sci Med Sci. 2013 Mar 1;68(3):271–8.	Constipation was not measured
Nishtala PS, Hilmer SN, McLachlan AJ, Hannan PJ, Chen TF. Impact of residential medication management reviews on drug burden index in aged-care homes: a retrospective analysis. *Drugs Aging*. 2009;26(8):677–686.	Constipation was not measured
Jamsen KM, Bell JS, Hilmer SN, et al. Effects of Changes in Number of Medications and Drug Burden Index Exposure on Transitions Between Frailty States and Death: The Concord Health and Ageing in Men Project Cohort Study. *J Am Geriatr Soc*. 2016;64(1):89–95.	Constipation was not measured
Ailabouni N, Mangin D, Nishtala PS. DEFEAT-polypharmacy: deprescribing anticholinergic and sedative medicines feasibility trial in residential aged care facilities. *Int J Clin Pharm*. 2019;41(1):167–178.	Constipation was not measured
Yeh YC, Liu CL, Peng LN, Lin MH, Chen LK. Potential benefits of reducing medication-related anticholinergic burden for demented older adults: a prospective cohort study. *Geriatr Gerontol Int*. 2013;13(3):694–700.	Constipation was not measured
Hsu WH, Wen YW, Chen LK, Hsiao FY. Comparative Associations Between Measures of Anti-cholinergic Burden and Adverse Clinical Outcomes. *Ann Fam Med*. 2017;15(6):561–569.	Constipation was not measured
Hochman MJ, Kamal AH, Wolf SP, et al. Anticholinergic Drug Burden in Noncancer Versus Cancer Patients Near the End of Life [published correction appears in J Pain Symptom Manage. 2019 May;57(5):e9-e10]. *J Pain Symptom Manage*. 2016;52(5):737–743.e3.	Constipation was not measured
Van der Meer HG, Wouters H, Pont LG, Taxis K. Reducing the anticholinergic and sedative load in older patients on polypharmacy by pharmacist-led medication review: a randomised controlled trial. *BMJ Open*. 2018;8(7):e019042.	Constipation was not measured
Pasina L, Djade CD, Lucca U, et al. Association of anticholinergic burden with cognitive and functional status in a cohort of hospitalized elderly: comparison of the anticholinergic cognitive burden scale and anticholinergic risk scale: results from the REPOSI study. *Drugs Aging*. 2013;30(2):103–112.	Constipation was not measured

Tiisanoja A, Syrjälä AM, Komulainen K, et al. Anticholinergic burden and dry mouth among Finnish, community-dwelling older adults. *Gerodontology*. 2018;35(1):3–10.	Constipation was not measured
Clark K, Lam LT, Agar M, Chye R, Currow DC. The impact of opioids, anticholinergic medications and disease progression on the prescription of laxatives in hospitalized palliative care patients: a retrospective analysis. *Palliat Med*. 2010;24(4):410–418.	Mostly acute opioid induced constipation was assessed
Gustafsson M, Lämås K, Isaksson U, Sandman PO, Lövheim H. Constipation and laxative use among people living in nursing homes in 2007 and 2013. *BMC Geriatr*. 2019;19(1):38.	No anticholinergic scale was utilized.
Valladales-Restrepo LF, Paredes-Mendoza M, Machado-Alba JE. Potentially inappropriate prescriptions for anticholinergic medications for patients with constipation [published online ahead of print, 2020 Mar 6]. *Dig Dis*. 2020;10.1159/000506981.	No association assessment between anticholinergic burden and constipation
