# Supplementary material for: Association between Anticholinergic Burden and Constipation: A Systematic Review
Source: Healthcare (Basel). 2021 May 13;9(5):581. doi: 10.3390/healthcare9050581 (PMC8153334; doi:10.3390/healthcare9050581)
Supplement: Supplementary file 1 [file healthcare-09-00581-s001.zip › healthcare-1174593 Appendix and Supplementary author proofreading done/Supplementary File S4.pdf]

Association between anticholinergic burden and constipation: a systematic review.

Supplemental file S4. Quality assessment of included studies.

Rob 2.0. A revised tool to assess risk of bias in randomized trials.

As Agar M, 2009 is a post-hoc analysis of a previously published clinical trial (Abernethy 2006), the original clinical trial was studied for quality assessment.

Bias domain and signalling question.	Response options		
	Lower risk of bias	Higher risk of bias	Other
**Bias arising from the randomisation process**			
1.1 Was the allocation sequence random?	Y		
1.2 Was the allocation sequence concealed until participants were enrolled and assigned to interventions?		N	
1.3 Did baseline differences between intervention groups suggest a problem with the randomisation process?	N		
Risk-of-bias judgment (low/high/some concerns)	**High risk**		
**Bias due to deviations from intended interventions**			
2.1 Were participants aware of their assigned intervention during the trial?		Y	
2.2 Were carers and people delivering the interventions aware of participants' assigned intervention during the trial?		Y	
2.3 If Y/PY/NI to 2.1 or 2.2: Were there deviations from the intended intervention that arose because of the trial context?	PN		
2.4 If Y/PY/NI to 2.3: Were these deviations likely to have affected the outcome?			NA
2.5 If Y/PY to 2.4: Were these deviations from intended intervention balanced between groups?			NA
2.6 Was an appropriate analysis used to estimate the effect of assignment to intervention?		PN	

2.7 If N/PN/NI to 2.6: Was there potential for a substantial impact (on the result) of the failure to analyse participants in the group to which they were randomized?	PN		
Risk-of-bias judgment (low/high/some concerns)	**Some concerns**		
**Bias due to missing outcome data**			
3.1 Were data for this outcome available for all, or nearly all, participants randomised?		N	
3.2 If N/PN/NI to 3.1: Is there evidence that the result was not biased by missing outcome data?		N	
3.3 If N/PN to 3.2: Could missingness in the outcome depend on its true value?		PN	
3.4 If Y/PY/NI to 3.3: Is it likely that missingness in the outcome depended on its true value?			NA
Risk-of-bias judgment (low/high/some concerns)	**Some concerns**		
**Bias in measurement of the outcome**			
4.1 Was the method of measuring the outcome inappropriate?	N		
4.2 Could measurement or ascertainment of the outcome have differed between intervention groups?	PY		
Risk-of-bias judgment (low/high/some concerns)	**High risk**		
**Bias in selection of the reported result**			
5.1 Were the data that produced this result analysed in accordance with a prespecified analysis plan that was finalised before unblinded outcome data were available for analysis?		PN	
Is the numerical result being assessed likely to have been selected, on the basis of the results, from:			
5.2 ... multiple eligible outcome measurements (eg, scales, definitions, time points) within the outcome domain?	PN		
5.3 ... multiple eligible analyses of the data?	PY		
Risk-of-bias judgment (low/high/some concerns)	**High risk**		
**Overall bias**			
Risk-of-bias judgment (low/high/some concerns)	**High risk**		

Y=Yes; PY=Probably Yes; PN=Probably No; N=No; NA=Not Applicable; NI=No Information.

JBI Critical Appraisal Checklist For Analytical Cross Sectional Studies.

	Briet J. 2017	De Vreese L. P. 2018	O'Connell J. 2018.	O'Dwyer M. 2016	Sevilla-Sánchez D. 2018	Mayer T. 2017
1. Were the criteria for inclusion in the sample clearly defined?	Y	Y	Y	Y	Y	Y
2. Were the study subjects and the setting described in detail?	N	N	N	N	Y	Y
3. Was the exposure measured in a valid and reliable way?	Y	Y	Y	N	Y	Y
4. Were objective, standard criteria used for measurement of the condition?	Y	Y	Y	Y	Y	Y
5. Were confounding factors identified?	N	Y	Y	Y	N	N
6. Were strategies to deal with confounding factors stated?	NA	Y	Y	Y	NA	NA
7. Were the outcomes measured in a valid and reliable way?	Y	N	N	N	UN	N
8. Was appropriate statistical analysis used?	Y	Y	Y	Y	Y	Y
Overall appraisal: (include/exclude/Seek further info)	Include	Include	Include	Include	Include	Include

Y=Yes; PY=Probably Yes; PN=Probably No; N=No; NA=Not Applicable; NI=No Information.

JBI Critical Appraisal Checklist For Cohort Studies

	Kuang-Hua H. 2012	Hwang S. 2019.	Allen C. 2017	Wawruch M. 2011
1. Were the two groups similar and recruited from the same population?	UN	Y	UN	Y
2. Were the exposures measured similarly to assign people to both exposed and unexposed groups?	Y	Y	Y	Y
3. Was the exposure measured in a valid and reliable way?	Y	Y	Y	Y
4. Were confounding factors identified?	N	Y	N	N
5. Were strategies to deal with confounding factors stated?	NA	Y	NA	NA
6. Were the groups/participants free of the outcome at the start of the study?	Y	UN	N	N
7. Were the outcomes measured in a valid and reliable way?	Y	Y	Y	Y
8. Was the follow up time reported and sufficient to be long enough for outcomes to occur?	Y	Y	Y	Y
9. Was follow up complete, and if not, were the reasons to loss to follow up described and explored?	Y	Y	UN	UN
10. Were strategies to address incomplete follow up utilized?	Y	NA	UN	N
11. Was appropriate statistical analysis used?	Y	Y	UN	Y
Overall appraisal: (include/exclude/Seek further info)	Include	Include	Include	Include

Y=Yes; PY=Probably Yes; PN=Probably No; N=No; NA=Not Applicable; NI=No Information.

Appendix references:

1. Abernethy AP, Currow DC, Hunt R, Williams H, Roder-Allen G, Rowett D, et al. A pragmatic 2 x 2 x 2 factorial cluster randomized controlled trial of educational outreach visiting and case conferencing in palliative care-methodology of the Palliative Care Trial [ISRCTN 81117481]. Contemp Clin Trials. 2006;27(1):83-100.
2. Huang K, Chan F, Shih H, Lee C. Relationship between potentially inappropriate anticholinergic drugs (PIADs) and adverse outcomes among elderly patients in Taiwan. J Food Drug Anal. 2012;20(4):930–7.
3. Agar M, Currow D, Plummer J, Seidel R, Carnahan R, Abernethy AP. Changes in anticholinergic load from regular prescribed medications in palliative care as death approaches. Palliat Med. 2009;23(3):257–65.
4. Wawruch M, Macugova A, Kostkova L, Luha J, Dukat A, Murin J, et al. The use of medications with anticholinergic properties and risk factors for their use in hospitalised elderly patients. Pharmacoepidemiol Drug Saf. 2012;21(2):170–6.
5. O'Dwyer M, Maidment ID, Bennett K, Peklar J, Mulryan N, McCallion P, et al. Association of anticholinergic burden with adverse effects in older people with intellectual disabilities: an observational cross-sectional study. Br J Psychiatry. 2016;209(6):504–10.
6. Allen C, Zarowitz BJ, O'Shea T, Datto C, Olufade T. Clinical and Functional Characteristics of Nursing Facility Residents with Opioid-Induced Constipation. Consult Pharm. 2017;32(5):285–98.
7. Briet J, Javelot H, Heitzmann E, Weiner L, Lameira C, D'Athis P, et al. The anticholinergic impregnation scale: Towards the elaboration of a scale adapted to prescriptions in French psychiatric settings. Therapie. 2017;72(4):427-37.
8. Mayer T, Meid AD, Saum KU, Brenner H, Schöttker B, Seidling HM, et al. Comparison of Nine Instruments to Calculate Anticholinergic Load in a Large Cohort of Older Outpatients: Association with Cognitive and Functional Decline, Falls, and Use of Laxatives. Am J Geriatr Psychiatry. 2017;25(5):531–40.
9. De Vreese LP, Mantesso U, De Bastiani E, Marangoni A, Weger E, Gomiero T. Anticholinergic burden in adult and elderly people with intellectual disabilities: Results from an Italian multicenter cross-sectional study. PLoS One. 2018;13(10):e0205897.

10. O'Connell J, Burke É, Mulryan N, O'Dwyer C, Donegan C, McCallion P, et al. Drug burden index to define the burden of medicines in older adults with intellectual disabilities: An observational cross-sectional study. Br J Clin Pharmacol. 2018;84(3):553-67.
11. Sevilla-Sánchez D, Molist-Brunet N, González-Bueno J, Solà-Bonada N, Espaulella-Panicot J, Codina-Jané C. Prevalence, risk factors and adverse outcomes of anticholinergic burden in patients with advanced chronic conditions at hospital admission. Geriatr Gerontol Int. 2018;18(8):1159–65.
12. Hwang S, Jun K, Ah YM, Han E, Chung JE, Lee JY. Impact of anticholinergic burden on emergency department visits among older adults in Korea: A national population cohort study. Arch Gerontol Geriatr. 2019;85:103912.
