# Supplementary material for: Association between Anticholinergic Burden and Constipation: A Systematic Review
Source: Healthcare (Basel). 2021 May 13;9(5):581. doi: 10.3390/healthcare9050581 (PMC8153334; doi:10.3390/healthcare9050581)
Supplement: Supplementary file 1 [file healthcare-09-00581-s001.zip › healthcare-1174593 Appendix and Supplementary author proofreading done/Supplementary Table S1.pdf]

Association between anticholinergic burden and constipation: a systematic review.

Supplementary Table S1. Alternate causes of constipation.

Study. Year	Physical activity	Fiber / Fluid intake	Polypharmacy	Opioids use	Diseases causing constipation
Agar M. 2009	No data. An overall low physical activity could be assumed on a palliative care program.	-	-	Opioids were the most common ACH drug.	-
Wawruch M. 2011	No data. An overall low physical activity could be assumed in a hospital setting.	-	The average number of drugs was: At admission: 5.8 ± 3.1; At discharge: 7.6 ± 2.9	Tramadol usage: At admission: 5% (n = 81); At discharge: 7.8% (n = 128)	6.22% of patients suffered from Parkinson's disease.
Kuang-Hua H. 2012	-	-	The average number of drugs was higher in patients with ACB: 4.34 vs 2.29.	-	-
O'Dwyer M. 2016	-	-	53.7% (n = 395) of patients were exposed to polypharmacy.	-	-
Allen C. 2017	-	-	-	100% of patients were prescribed opioids	-
Briet J. 2017	-	-	-	-	-
Mayer T. 2017	-	-	-	-	-
De Vreese L. P. 2018	-	-	13.4% (n= 37) of patients, with a mean daily consumption of 7.3 ± 2.4 drugs	-	-
O'Connell J. 2018	No data. An overall low physical activity could be assumed in a highly dependent population.	-	62.2% (n = 421) of patients. The median number of drugs was 6.0 (IQR 6.0).	-	-
Sevilla-Sánchez D. 2018	No data. An overall low physical activity could be assumed in a palliative hospital setting.	-	A higher number of drugs were prescribed in patients receiving ACH drugs and in high ACB patients: 5 ± 3.4 vs 9.8 ± 3.6 and 11.1 ± 3.6 drugs respectively.	-	-
Hwang S. 2019	-	-	Polypharmacy was more prevalent in patients with ACB: 40.7% (n= 24,144) vs 25.3% (n= 14,994)	-	-

Polypharmacy was defined as >5 drugs; ACH: Anticholinergic, ACB: Anticholinergic Burden.
